# *LRRC6* Mutation Causes Primary Ciliary Dyskinesia with Dynein Arm Defects

**DOI:** 10.1371/journal.pone.0059436

**Published:** 2013-03-19

**Authors:** Amjad Horani, Thomas W. Ferkol, David Shoseyov, Mollie G. Wasserman, Yifat S. Oren, Batsheva Kerem, Israel Amirav, Malena Cohen-Cymberknoh, Susan K. Dutcher, Steven L. Brody, Orly Elpeleg, Eitan Kerem

**Affiliations:** 1 Department of Pediatrics, Washington University School of Medicine, St. Louis, Missouri, United States of America; 2 Department of Cell Biology and Physiology, Washington University School of Medicine, St. Louis, Missouri, United States of America; 3 Department of Pediatrics, Hadassah Hebrew University Medical Center, Jerusalem, Israel; 4 Department of Medicine, Washington University School of Medicine, St. Louis, Missouri, United States of America; 5 Department of Genetics, The Hebrew University, Jerusalem, Israel; 6 Department of Pediatrics, Ziv Medical Center, Safed, Israel; 7 Department of Genetics, Washington University School of Medicine, St. Louis, Missouri, United States of America; 8 Monique and Jacques Roboh Department of Genetic Research, Hadassah Hebrew University Medical Center, Jerusalem, Israel; The Children’s Hospital of Philadelphia, United States of America

## Abstract

Despite recent progress in defining the ciliome, the genetic basis for many cases of primary ciliary dyskinesia (PCD) remains elusive. We evaluated five children from two unrelated, consanguineous Palestinian families who had PCD with typical clinical features, reduced nasal nitric oxide concentrations, and absent dynein arms. Linkage analyses revealed a single common homozygous region on chromosome 8 and one candidate was conserved in organisms with motile cilia. Sequencing revealed a single novel mutation in *LRRC6* (Leucine-rich repeat containing protein 6) that fit the model of autosomal recessive genetic transmission, leading to a change of a highly conserved amino acid from aspartic acid to histidine (Asp146His). LRRC6 was localized to the cytoplasm and was up-regulated during ciliogenesis in human airway epithelial cells in a Foxj1-dependent fashion. Nasal epithelial cells isolated from affected individuals and shRNA-mediated silencing in human airway epithelial cells, showed reduced LRRC6 expression, absent dynein arms, and slowed cilia beat frequency. Dynein arm proteins were either absent or mislocalized to the cytoplasm in airway epithelial cells from a primary ciliary dyskinesia subject. These findings suggest that LRRC6 plays a role in dynein arm assembly or trafficking and when mutated leads to primary ciliary dyskinesia with laterality defects.

## Introduction

Motile cilia and flagella are essential, highly conserved organelles that extend from the cell to perform specialized functions, including motility and propulsion, and are present in the upper and lower respiratory tract, brain ventricles, and reproductive organs. A motile cilium is composed of an axoneme containing nine outer microtubule doublets and an inner central pair. The outer doublets are associated with dynein motor proteins, organized as outer dynein arms (ODA) and inner dynein arms (IDA). These proteins allow adjacent outer doublets to slide against one other and thus provide movement. Nexin links tether and limit the motion of microtubular doublets, and radial spokes control dynein arm activity relaying signals from the central microtubular pair to the dynein arms [1]. As a vital component of the mucociliary apparatus, cilia are critical for respiratory tract host defense [2], and when dysfunctional, may lead to primary ciliary dyskinesia (PCD) (CILD1: MIM 244400). PCD is a rare, genetically heterogeneous disorder, which is usually inherited as an autosomal recessive trait, and is caused by mutations in genes that code for the dynein proteins or regulatory factors affecting those proteins [1]–[3]. These genetic defects can render the cilia immotile or lead to an abnormal beating pattern [4]. Impaired mucociliary clearance in affected individuals may result in acute and chronic infections of the lung, middle ear, and paranasal sinuses [1]–[3]. Furthermore, cilia defects in the embryonic node during development cause laterality defects, such as *situs inversus totalis* or heterotaxy, in approximately half of PCD cases [5]. Ciliary dysmotility can also cause infertility and has been linked to prenatal hydrocephalus [6], [7].

Our understanding of the link between genetic defects and ultrastructural changes of cilia has greatly advanced over the past decade. Owing to conservation of cilia and flagellar structures, studies of these organelles in model organisms, from algae (*Chlamydomonas reinhardtii*) to zebrafish (*Danio rerio*) to mammals, have provided insights into structure, function, and genetics of the human cilium. Thus far, studies using these organisms and others have led to the identification of sixteen different genes that when mutated produce unambiguous clinical phenotypes of PCD in humans. These genes include *DNAH5* (MIM 603335), *DNAI1* (MIM 604366), *DNAI2* (MIM 605483), *TXNDC3* (MIM 607421), *DNAL1* (MIM 610062), *DNAH11* (MIM 603339), *HEATR2* (MIM 614864), *DNAAF1* (MIM 612517), *DNAAF2* (MIM 613190), *DNAAF3* (MIM 614566), *RSPH4A* (MIM 612647), *RSPH9* (MIM 612648), *CCDC39* (MIM 613798), *CCDC40* (MIM 613799), *CCDC103* (MIM 614677) and *HYDIN* (MIM 610812 ) [8]–[25]. Several genes, *DNAAF1*, *DNAAF2*, *DNAAF3* and *HEATR2*, encode proteins that are involved in dynein arm assembly while the others are essential structural components of the ciliary axoneme. Nonetheless, mutations in these genes still account for less than half of all PCD cases, and our understanding of the critical components of cilia assembly is incomplete [1], [6].

Here, we describe a single non-synonymous mutation in *LRRC6* that causes PCD in several members of two unrelated, consanguineous Palestinian families. *LRRC6* is evolutionarily conserved across the phylogenetic tree, and is found in mammals, zebrafish (*D. rerio*), flies (*Drosophila melanogaster*), protozoa (*Trypanosoma brucei*), algae (*C. reinhardtii*), but not in worms (*Caenorhabditis elegans*). There are fourteen other proteins with leucine-rich repeats (LRR) in the cilia proteome [26]. The LRR region in LRRC6 most closely resembles that of the SDS22-like subfamily of LRR proteins [27], a set of proteins with diverse functions, including splicing factors and nuclear export proteins [28]. Airway epithelial cells isolated from affected individuals had reduced LRRC6 expression, axonemal defects with mislocalized dynein proteins, and markedly slowed cilia beat frequency, effects that were all recapitulated by shRNA-mediated knockdown of *LRRC6* in normal airway epithelial cells.

## Methods

### Patients

**Figure 1:**
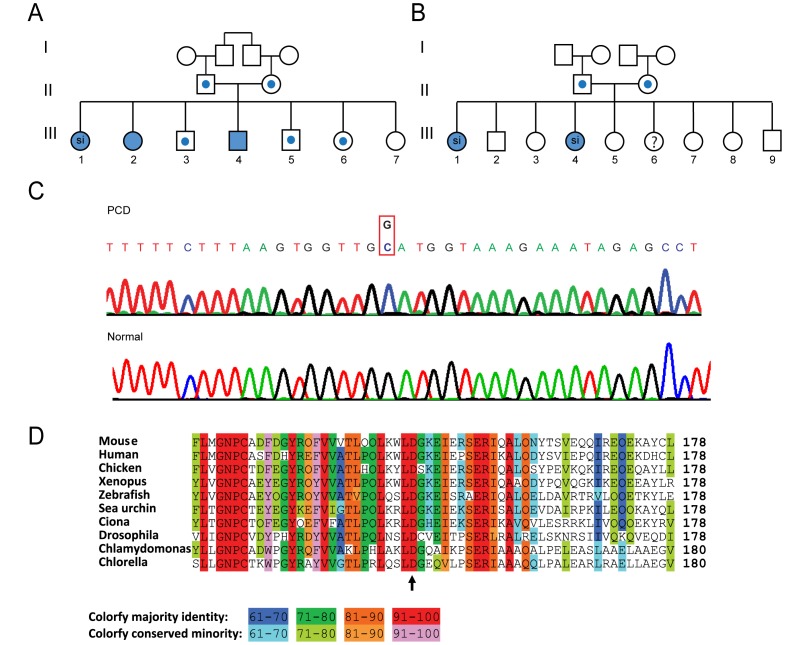
Family pedigree and genetic analyses. Pedigree of consanguineous kindred from two unrelated families in Palestinian communities (**A and B**). Solid symbols: affected individuals; central dots represent heterozygous individuals; Abbreviations: **si**, *situs inversus totalis.* Chromatogram showing the nucleotide sequence (**C**) of the *LRRC6* Exon 5 adjacent to the mutation site, which resulted in G-to-C change at base position, c.436 (Chr8∶133645203). Amino acid sequence of the LRRC6 protein around the mutated residue. Note the high degree of conservation in diverse organisms that have motile cilia or flagella (**D**).

Subjects with clinical features consistent with PCD from two unrelated, endogamous families were studied (**Figure 1A** **and** **Table 1**).

**Table 1:** Clinical characteristics of PCD subjects with *LRRC6* mutant alleles.

Patient	Age (years)	Gender	Clinical manifestations	Laterality	Ultrastructural defects	Nasal NO (ppb)
A III-1	28	F	OM, RS, BR	SI	ODA – IDA	46.7
A III-2	25	F	OM, BR, RS	SS	ODA – IDA	10.1
A III-4	15	M	OM, RS, BR	SS	ODA – IDA	31.4
B III 1	21	F	OM, BR	DC	ODA – IDA	26.4
B III 4	13	F	OM, BR	DC	ODA – IDA	ND

**Abbreviations:** BR, bronchiectasis; DC: dextrocardia; OM, chronic or recurrent otitis media; RS, rhinosinusitis; SI, *situs inversus totalis*; SS, *situs solitus*; ODA, outer dynein arm; IDA, inner dynein arm; ND, not done.

### Ethics Statement

All individuals or their parents provided written informed consent for diagnostic evaluation and genetic characterization. The study protocol was approved by the Hadassah-Hebrew University Human Subjects Committee. Institutional approval was obtained to conduct both human and animal research. Anonymized human airway epithelial cells from surgical excess of large airways that were trimmed during the transplant procedure, of lung donated for transplantation at Washington University in St. Louis were also used in these studies. Research using cells originating from deidentified cadaver specimen (surgical excess of large airways of lung) is exempt from regulation and is not governed by NIH regulation 45 CFR Part 46.

### Genetic Analyses and Sequencing

Genetic linkage analysis was performed on three affected members (III-1, III-2 and III-4 in **Figure** **1A**) using the GeneChip Human Mapping 250 K Nsp Affymetrix Array as previously described [29]. The sequence of LRRC6 twelve exons and their flanking intronic regions were analyzed by forward and reverse Sanger dideoxy sequencing using the appropriate primers.

### Airway Epithelial Cells

Nasal epithelial cells from subjects were obtained from the inferior turbinate by cytology brush [30]. Human airway epithelial cells were isolated from surgical excess of large airways (tracheobronchial segments) that were trimmed during the transplant procedure, of lungs donated for transplantation. Cells were expanded in culture, seeded on supported membranes (Transwell, Corning Inc., Corning, NY), and re-differentiated using air-liquid interface conditions [31]. Cell preparations were maintained in culture for four to ten weeks.

### Gene Silencing of Airway Epithelial Cells

shRNA targeted sequences generated by the Children’s Discovery Institute shRNA Library Core, were inserted into pLKO.1 lentivirus vectors that includes a U3 promoter and a puromycin resistant cassette. The shRNA sequences used were: GCCCAAGGTAGGAGAAGTAAT (shRNA#1), GAACACAACGACTGT GTCATT (shRNA#2), GATCTCAGACAACGGGTCATT (shRNA#3), CCTGTTTGTTTACTCCT GAAT (shRNA#4) and CCTAAATGTGAATGAGCCCAA (shRNA#5). A non-targeted sequence with a yellow fluorescent protein (YFP) reporter (a gift from Y. Feng and G.D. Longmore), was used as control [32]. Undifferentiated airway epithelial cells were transfected and selected using established protocols [33], [34]. Briefly, vesicular stomatitis virus envelope glycoprotein (VSV-G)-pseudotyped vectors were generated by three-plasmid cotransfection of HEK 293T cells using Fugene 6 (Roche, WI). The generated viral supernatant was collected, filtered and used to infect airway epithelial cells. These cells were then selected by adding puromycin to the culture media. Once confluent, airway epithelial cells were grown at an air-liquid interface.

### Epithelial Cell Immunofluorescent Staining and Immunoblot Analyses

Normal human lung obtained from excess tissue donated for lung transplantation was fixed, immunostained and imaged as previously described [31], [35]. Human tracheobronchial epithelial cells (hTEC) collected from non-PCD subjects and differentiated at an air-liquid interface [31] were similarly examined for protein expression using primary antibodies against LRRC6 (1∶100, HPA028058/SAB2103053, Sigma Aldrich, MO), acetylated α-tubulin (1∶5000, clone 6-11-B1, Sigma Aldrich), LAMP2 (1∶200, Abcam, Cambridge, MA), EEA1 (1∶100, BD Biosciences, San Jose, CA), χ-tubulin (1∶500, Clone Gtu-88, Sigma-Aldrich), DNAH7 (1∶50, Novus Biologicals, Littleton, CO), and DNAI1 (1∶5000, gift from Dr. Lawrence Ostrowski, University of North Carolina, Chapel Hill, NC [36]) which were detected using secondary antibodies conjugated to Alexa Fluor dyes (A-21202, A-21206, A-31570 and A-31572; Life Technology, Grand Island, NY). Nuclei were stained using 4′, 6-diamidino-2-phenylindole 1.5 µg/mL. Images were acquired using epifluorescent microscopy and adjusted globally using Photoshop (Adobe Systems, San Jose, CA). Cells were imaged and recorded as previously described [31], [35]. For immunoblot analyses, cell supernatants were resolved by SDS-PAGE (7.5%) then transferred to PVDF membranes. The immunoblots were blocked, incubated with anti-LRRC6 (1∶100 dilution, SAB1407241, Sigma-Aldrich) or anti-Foxj1 antibody (1∶300), and detected with enhanced chemiluminescence using established protocols [35].

### RT-PCR Analyses

RNA expression was assessed by RT-PCR amplification using the following oligonucleotide primer sets: human *LRRC6*, 5′-GCAGGCTTTGATGGACGTTG and 5′-GCCTGTAGGTGGTCTTTGCT; murine *LRRC6*, 5′-AAGTTGACCCCAGCAAGCAT and 5′-CTCACTGGGTTCATCTCGGG; *Foxj1*, 5′-CCCGACGACGTGGACTAC and 5′-GGCGGAA GTAGCAGAAGTTG; *DNAI1*, 5′-AACGACGGCTGTCCCTAAAG and 5′-AGCCTACAAAACGC TCCCTC; and DNAH7, 5′-ACTTGCAGAATCGCATCCCA and 5′-CTCCTCTCCGCTC ACTTGTC, and detected using SYBR green in Lightcycler 480 (Roche, Indianapolis, IN) [37]. Briefly, RNA was isolated from cells using an Illustra RNAspin kit (GE Healthcare, Buckinghamshire, UK). RNA was reverse transcribed using a cDNA Reverse Transcription Kit, and then amplified using the TaqMan Fast Universal PCR Master Mix (both from Applied Biosystems, Carlsbad, CA). Gene expression was normalized to glyceraldehyde 3-phosphate dehydrogenase expression.

### Airway Epithelial Cell Videomicroscopy and Electron Microscopy

Nasal epithelial cells collected from subjects with PCD were examined using previously published protocols [38]. Videomicroscopy of ciliated epithelial cells was performed using an inverted microscope with a 20X phase contrast objective (Eclipse Ti-U; Nikon, Melville, NY) enclosed in a customized environmental chamber maintained at 37°C. Images were captured by a high-speed video camera and processed with the Sisson-Ammons Video Analysis system (Ammons Engineering, Mt. Morris, MI, USA) and analyzed using established methodologies [13], [39]. Cilia beat frequency was analyzed in at least five fields obtained from each cell preparation. Patient samples were prepared for electron microscopy using previously published protocols; a minimum of 10 ciliary axoneme cross-sections were reviewed and examined in a blinded fashion to define ultrastructure using established criteria [17]. For shRNA treated samples, more than 100 axonemes were blindly reviewed by investigators and scored for ultrastructural defects [13].

### Statistical Analyses

Data are expressed as mean ± standard deviation (SD). Statistical comparisons between groups were made using single factor analysis of variance (ANOVA) with Bonferroni correction for multiple comparisons. Individual comparisons were made using Student Two-tail test.

## Results and Discussion

Five subjects with clinical features consistent with PCD, including chronic sinusitis, bronchiectasis, recurrent otitis media and laterality defects, from two unrelated, consanguineous Palestinian families were studied (**Figure 1A** **and** **Table 1**). Subjects had reduced nasal nitric oxide concentrations; a finding associated with PCD and is suggested as a screening tool [38], [40], [41]. No ciliary motion compared to healthy controls (**Supplementary video S1 and S2**) and absent dynein arms (mean ODA and IDA numbers: 0.3±0.4 and 0.4±0.6 per axoneme, respectively; n = 30 axonemal cross sections) were found in at least one of the affected siblings from each family. Analysis of single-nucleotide polymorphism (SNP) haplotype on three affected members in one family (III-1, III-2 and III-4 in **Figure** **1A**) revealed multiple regions of homozygosity in each individual, but all three shared a single homozygous genomic region on chromosome 8 (125.70–142.16 Mb, based on Human Genome build 19). Shared haplotype of STR markers that span the region (D8S1720, D8S256 and D8S1743) were noted in two affected siblings from a second family (III-1 and III-4 in **Figure 1B**). Within this common 16.45 Mb genomic region, 43 protein coding genes were present, and seven candidates [*LRRC6*, *KIAA0196* (MIM 610657), *EIF2C2* (MIM 606229), *NDRG1* (MIM 605262), *EFR3A* (MIM 611798), *EIF2C2* (606229), and *DDEF1* (MIM 605953)] were annotated in the ciliary proteome [26]. Only one gene was conserved across all organisms with motile cilia, and DNA sequencing revealed a single, novel, missense mutation that created a G-to-C change at base position c.436 (Chr8∶133645203) in exon 5 of *LRRC6* (leucine-rich repeat containing protein 6), which resulted in substitution of aspartic acid to histidine (Asp146His) (**Figure 1C**), an amino acid that is highly conserved in organisms with motile cilia and flagella (**Figure 1D**). The mutation segregated with disease in an autosomal recessive transmission. The five affected individuals were homozygous for the mutated allele whereas the parents and three unaffected siblings from the index family were heterozygous for the mutation**.** The mutation was not listed in dbSNP135, but was found on 3 of 13006 alleles from 6503 healthy individuals reported in the Exome Variant Server (http://evs.gs.washington.edu/EVS; Exome Variant Server, NHLBI Exome Sequencing Project (ESP), Seattle, WA).

*LRRC6* was originally identified as *LRTP* and expressed during spermatogenesis in mice and humans [42]. LRRC6 contains 6 N-terminal LRR repeats, an LRRcap domain and a CS-like domain near the C-terminus [43]. The (Asp146His) falls in the LRRcap domain, a sequence important for protein-protein interaction, regulation of RNA-binding specificity, and RNA nuclear export [44]. Expression of the *C. reinhardtii* orthologue was increased following deflagellation when compared to pretreatment values, consistent with transcriptional up-regulation of flagellar genes during cillogenesis [45]. The homologous gene in *D. rerio* (*Lrrc6l*), when mutated, results in ciliary motility defects ranging from immotility to disorganized beating in the pronephros and neural tube, but normal axonemal ultrastructure [27]. *D. melanogaster tilB* mutants have defective sperm flagella motility and dysfunctional ciliated dendrites of the chordotonal organs. Furthermore, these mutant sperm axoneme lacked dynein arms [46]. The LRRC6 orthologue, TbLRTP, of the *T. brucei* localizes to basal bodies and is critical for basal body duplication, flagellum assembly, and cytokinesis [47]. Altogether, these data indicate that the LRRC6 protein has conserved functions central to ciliary and flagellar processes.

**Figure 2:**
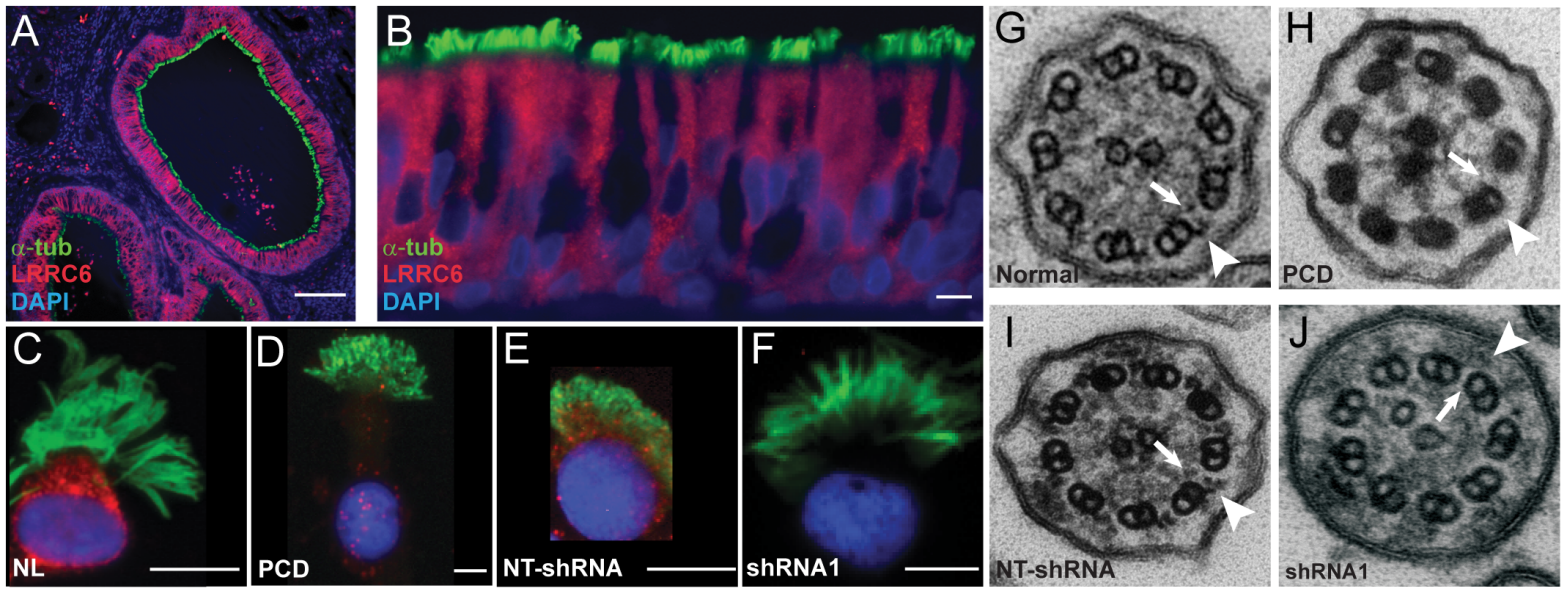
LRRC6 expression in ciliated airway epithelial cells. Photomicrographs of normal human lung section (**A**) (scale bar = 100 µm) and bronchial epithelium (**B**) following immunofluorescent staining for LRRC6, which reveals the cytoplasmic localization of the protein (LRRC6, red) only in ciliated cells (acetylated α-tubulin, a cilia marker, green; DAPI: blue) (scale bar = 10 µm). Immunofluorescent staining of nasal epithelial cells cultured at an air-liquid interface from a healthy subject demonstrating cytoplasmic localization of LRRC6 (**C**) as compared to decreased expression in a cell from a PCD subject (**D**). LRRC6 was similarly present in a non-targeted shRNA (NT) treated airway epithelial cells (**E**) but absent in *LRRC6-specific shRNA* transfected cells (**F**) (scale bar = 10 µm). Ultrastructural appearance of cilia from a normal control (**G**), PCD subject (**H**)**,** and airway epithelial cells following transfection with non-targeted (**I**) and *LRRC6-*targeted (**J**) shRNA sequences. Arrow and arrow-head indicate inner and outer dynein arms, respectively.

To better elucidate the function of LRRC6 in cilia assembly, we examined its expression in human airway epithelial cells. LRRC6 was not found in the ciliary axoneme, but was distributed throughout the cytoplasm of ciliated airway epithelial cells (**Figure 2A and 2B**), and localized proximally to basal bodies (**Supplementary Figure S1**), suggesting its involvement in assembly or trafficking during cilia biogenesis.

*LRRC6* expression was silenced using an RNAi approach in primary airway epithelial cells obtained from excess tracheal and bronchial tissue from healthy lung transplant donors to define its role in differentiation, cilia assembly, and function. *LRRC6* was reproducibly inhibited by each of the *LRRC6*-specific shRNA sequences when compared to cells transfected with non-targeted shRNA sequences as determined using both RT-PCR and immunoblot analyses (**Supplementary Figure S2A** and **S2B**). Cilia were present on the apical surface of cells treated with shRNA sequences and affected individuals, which showed that LRRC6 was not required for ciliogenesis (**Supplementary Figure S2C**). When compared to non-PCD or non-targeted shRNA transfected cells (**Figures 2C and 2E**, respectively), LRRC6 was markedly reduced in the cytoplasm of nasal epithelial cells from PCD subjects and *LRRC6*-specific shRNA transfected cells (**Figures 2D and 2F**). Furthermore, consistent with the axonemal defect observed in affected subjects (**Figure 2H**), ultrastructural analyses of cilia from silenced airway epithelial cells had truncated or absent dynein arms (**Figure 2J**) compared to normal and non-targeted shRNA transfected cells (**Figure 2G and 2I** **, respectively**).

**Figure 3:**
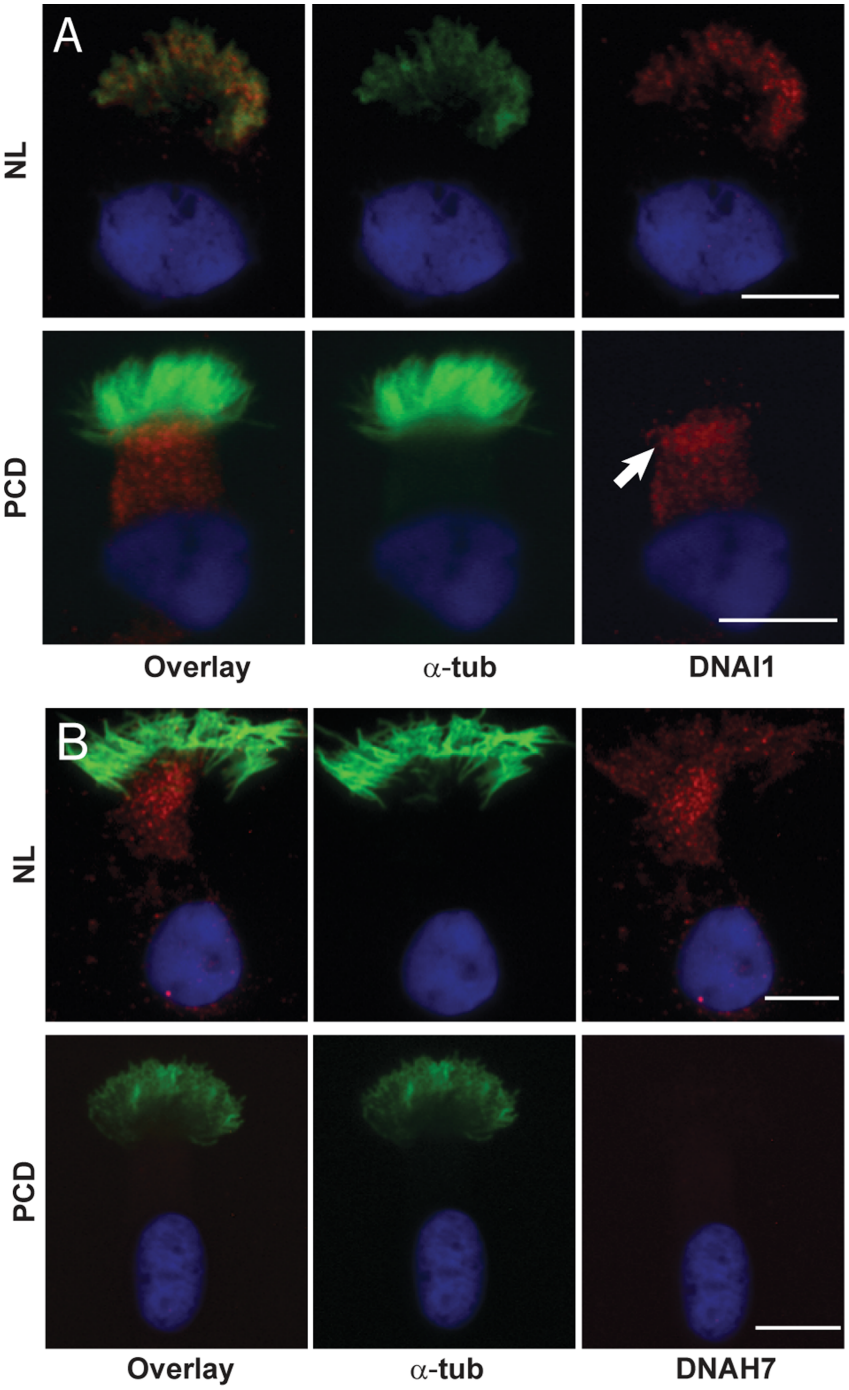
Inner and outer dynein protein mislocalization. Immunofluorescent staining of airway epithelial cells cultured at an air-liquid interface from normal (NL) and PCD subjects (PCD) for DNAI1 (red) (**A**), showing that outer dynein arm marker was localized to cilia of normal cells, but not in PCD cells. DNAI1 was present in the cytoplasm and most prominent beneath the ciliary axoneme in the PCD subject, suggesting mislocalization. The inner dynein arm marker, DNAH7 (red) (**B**), was localized to cilia of normal airway epithelial cells (NL) but absent in cells collected from PCD subjects (PCD). Immunofluorescent staining for DNAI1 or DNHA7 (red), α-tubulin (green) and DAPI (blue) (Scale bar = 10 µm).

**Figure 4:**
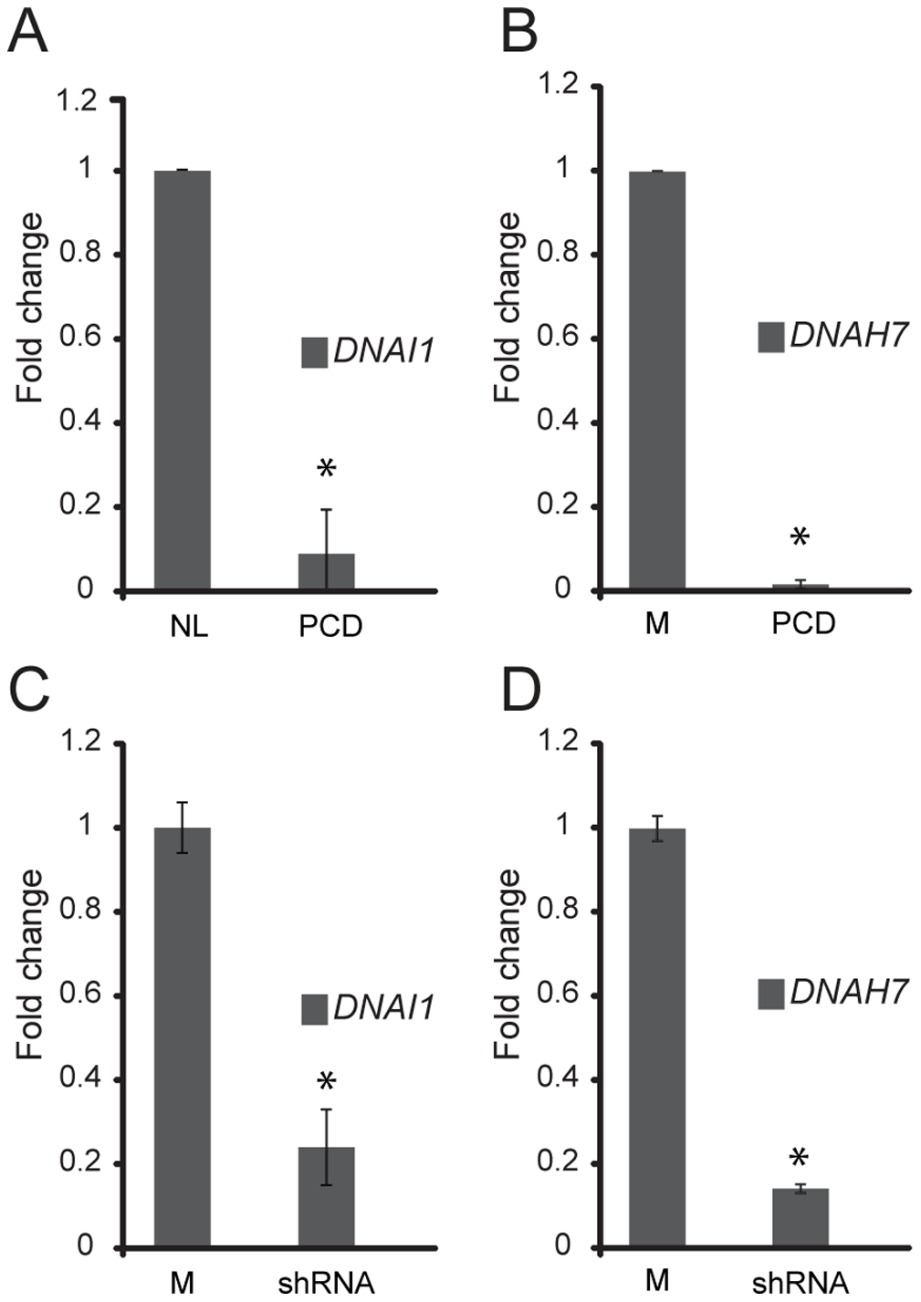
RT PCR analysis of *DNAI1* and *DNAH7* expression in PCD and RNAi silenced cells. *DNAI1* and *DNAH7* expression in nasal cells from PCD subjects (**A and B**) was markedly reduced as compared to cells from a healthy subject (NL) (n = 3 subjects, student t-test, p<0.001). Similarly, *DNAI1 and DNAH7* expression was decreased in *LRRC6*-specific shRNA transfected cells (**C and D**) compared to non-transfected control cells (M) (student t-test, p<0.001). (*) indicates a significant difference compared to control samples.

**Figure 5:**
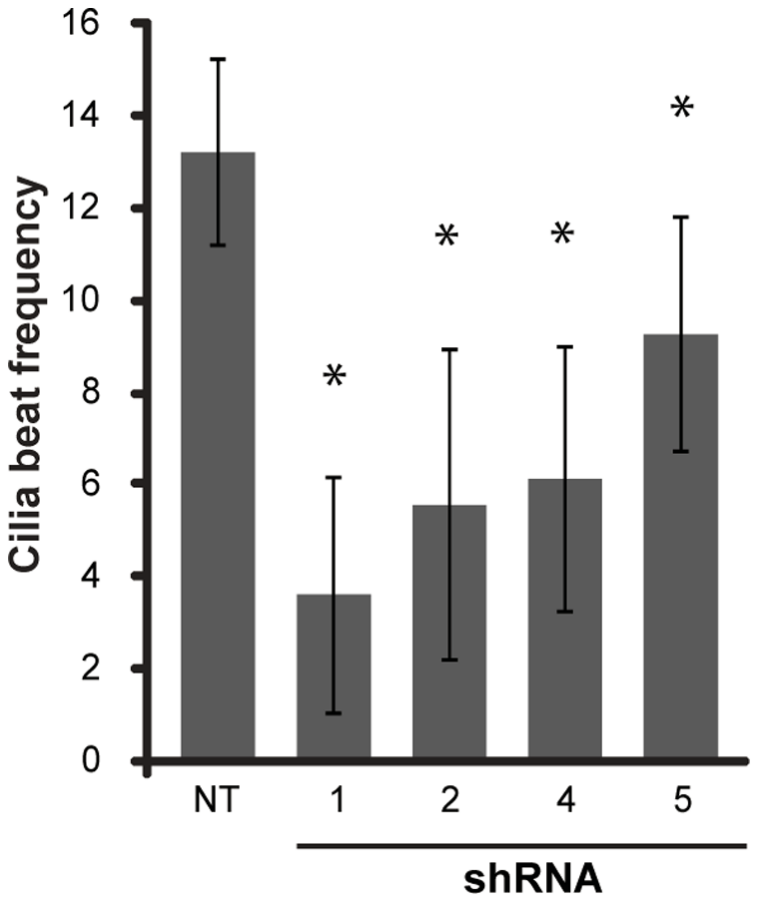
Cilia beat frequency. Mean cilia beat frequency in cells transfected with different shRNA targeted *LRRC6* sequences (n = 50 fields, ANOVA with Bonferroni correction, p<0.005). (*) indicates a significant difference compared to control samples.

To examine the role of LRRC6 in dynein arm assembly, we immunostained ciliated cells with antibodies against DNAI1, an outer dynein arm polypeptide, and DNAH7, an inner dynein arm polypeptide. Neither DNAI1 nor DNAH7 were detected in cilia from PCD subjects, but DNAI1 was found in the apical cytoplasm of the epithelial cell (**Figure 3A** **)** suggesting mislocalization of the protein and failure of axonemal transport. In contrast, DNAH7 was not detected in the PCD cells, which may be related to protein degradation or suppressed expression. The latter was further evaluated by examining the expression of *DNAI1* and *DNAH7* in nasal cells for PCD subjects. *DNAI1* and *DNAH7* transcription was markedly reduced in nasal cells from three PCD subjects (III-1, III-2 and III-4 in **Figure** **1A** **)** compared to a healthy control; findings that were also recapitulated in *Lrrc6*-specific shRNA targeted cells, suggesting that mutations in LRRC6 alters the expression of genes encoding some ODA and IDA proteins (**Figure 4**). While these findings indicate that LRRC6 is important for expression, trafficking, or assembly of normal dyneins, the pattern was also reminiscent of mislocalization of the ODA dynein DNAH5, previously described in PCD subjects with certain DNAH5 mutations [48], where mutations in DNAH5, hindered proper trafficking of ODA proteins into the ciliary axoneme and led to their accumulation in the cytoplasm. These findings further support the notion that ODA and possibly IDA proteins are assembled in the cytoplasm and are transported into the cilia axoneme as precursors. Furthermore, airway epithelial cells transfected with *LRRC6*-specific shRNA had markedly slower ciliary motion when compared to controls, as assessed using high-speed videomicroscopy (**Figure 5** **)**. Nasal cells collected from subjects with PCD had no cilia motion when examined using high speed videomicroscopy (**Supplementary Videos S2**) [38].

**Figure 6:**
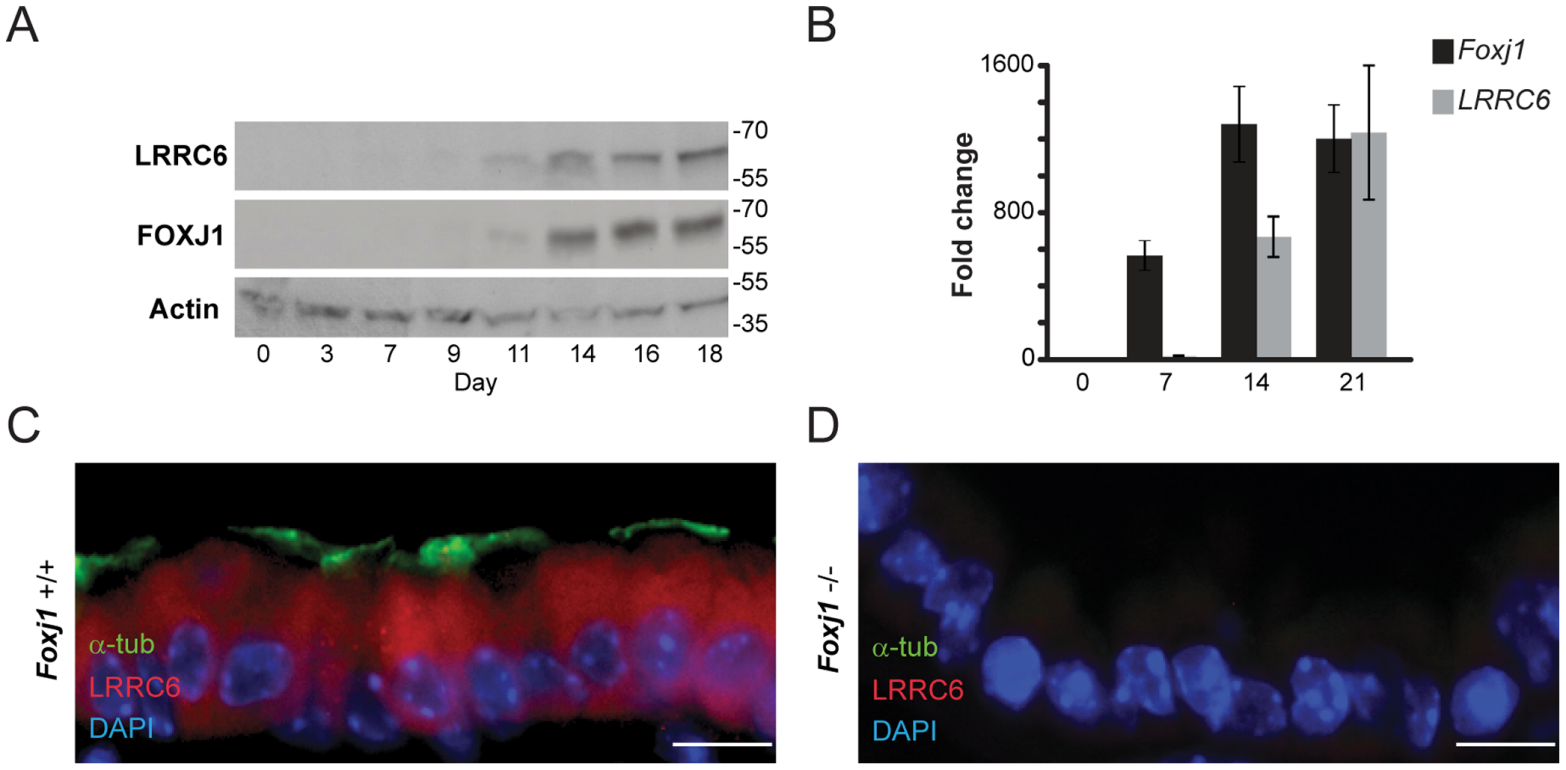
*LRRC6* relation to *Foxj1*. Immunoblot analysis of differentiating human tracheal epithelial cells collected from healthy subjects and grown at an air-liquid interface (**A**) showing LRRC6 paralleling Foxj1 expression. *LRRC6* expression measured using RT-PCR (**B**), increased significantly after the onset of ciliogenesis (ANOVA, p = 0.0004; individual student two-tail t-test, p = 0.003 and p = 0.001, comparing expression between day 0 and day 7, and between day 7 and day 14, respectively). *LRRC6* expression in tracheal airway epithelial cells isolated from wild-type mice (*Foxj1^+/+^*) (**C**), compared to cells from Foxj1-deficient littermates (*Foxj1^−/−^*) (**D**), showing that virtually no LRRC6 was detected in the cytoplasm of *Foxj1^−/−^* cells. Immunofluorescent staining for LRRC6 (red) and α-tubulin (green). (scale bar = 10 µm).

The relationship between *LRRC6* expression and ciliogenesis, was examined using primary culture of hTEC as previously described [31]. *LRRC6* was initially detected during early ciliary differentiation, which coincided with the expression of the master ciliogenesis gene, *Foxj1* **(** **Figure 6A and 6B** **)** [31]. This relationship was further established by assessing *Lrrc6* expression in airway epithelial cells isolated from syngeneic wild-type (*Foxj1^+/+^*) and *Foxj1*-deficient (*Foxj1^−/−^*) mice [49]. *Lrrc6* levels were markedly reduced in *Foxj1^−/−^* airway epithelial cells when compared to *Foxj1^+/+^* cells (**Figure 6C and 6D**), indicating that Foxj1 regulated *Lrrc6* expression.

In summary, we show that a mutation in *LRRC6*, Asp146His, caused PCD in affected individuals from two unrelated families, which resulted in axonemal defects of the dynein arms and ciliary dysmotility. The association between LRRC6 and PCD was also recently reported in European subjects, thus confirming the importance of LRRC6 in cilia structure and function [50]. The ultrastructural and functional phenotypes observed in our cohort were recapitulated in *LRRC6*-silenced human airway epithelial cells. Regulated by Foxj1, LRRC6 is expressed in the cytoplasm of normal ciliated airway epithelial cells and absent from the ciliary axoneme, indicating that it is not a structural protein, findings that are consistent with published proteomic analyses that did not detect LRRC6 in cilia [51], [52]. The absence of LRRC6 in cilia from these studies, and mislocalization of outer dynein, DNAI1, suggests a role in the preassembly of the dynein arms, like DNAAF1, DNAAF2, and DNAAF3, or their transport to the basal bodies, similar to ODA16 [14], [20], [25]. The novel finding of reduced expression of the outer and inner arm markers, DNAI1 and DNAH7, may also indicate that LRRC6 is involved in transcriptional regulation of some dynein proteins. Our findings are consistent with observations in other experimental models that conclusively show LRRC6 and its orthologues are involved in cilia assembly and function [50]. Thus, *LRRC6* can be added to the rapidly growing list of genes that when mutated cause PCD.

## Supporting Information

Figure S1 **Co-localization of LRRC6 with different organelles.** Immunofluorescent staining of tracheobronchial epithelial cells from healthy subject showing no co-localization of LRRC6 (red) with markers of endosomes (green), and lysosomes (green). However, LRRC6 localized with χ-tubulin, a marker for basal bodies (green). Nuclei were stained using DAPI (blue). acetylated α-tubulin, a cilia marker, is shown in turquoise (scale bar = 10 µm).
(TIF)

Figure S2 **RT PCR analysis of** ***LRRC6*** **expression in RNAi silenced cells.** **(A)** *LRRC6* expression in LRRC6-specific shRNA transfected airway epithelial cells **(B)** Immunoblot analyses of airway epithelial cells transfected with three different *LRRC6*-specific shRNA or non-targeted shRNA (NT) sequences and nontransfected control cells (M). **(C)** En face images of LRRC6 in cultured preparations of ciliated airway epithelial cells from a normal donor, transfected with either non-targeted, control shRNA (NT) or different *LRRC6* targeted shRNA sequences. LRRC6 (red), acetylated α-tubulin (green), a ciliated cell marker, and co-stained with DAPI (blue). (scale bar = 20 µm).
(TIF)

Video S1 **Healthy human nasal epithelial cells.** Nasal epithelial cells from a healthy non-PCD subject showing normal cilia motion.
(MP4)

Video S2 **Nasal epithelial cells from a subject with PCD.** Nasal epithelial cells from a PCD subject with the *LRRC6* mutation showing no cilia motion.
(MP4)
