# Supplementary figures and images for: LRRC6 Mutation Causes Primary Ciliary Dyskinesia with Dynein Arm Defects
Source: PLoS One. 2013 Mar 19;8(3):e59436. doi: 10.1371/journal.pone.0059436 (PMC3602302; doi:10.1371/journal.pone.0059436)

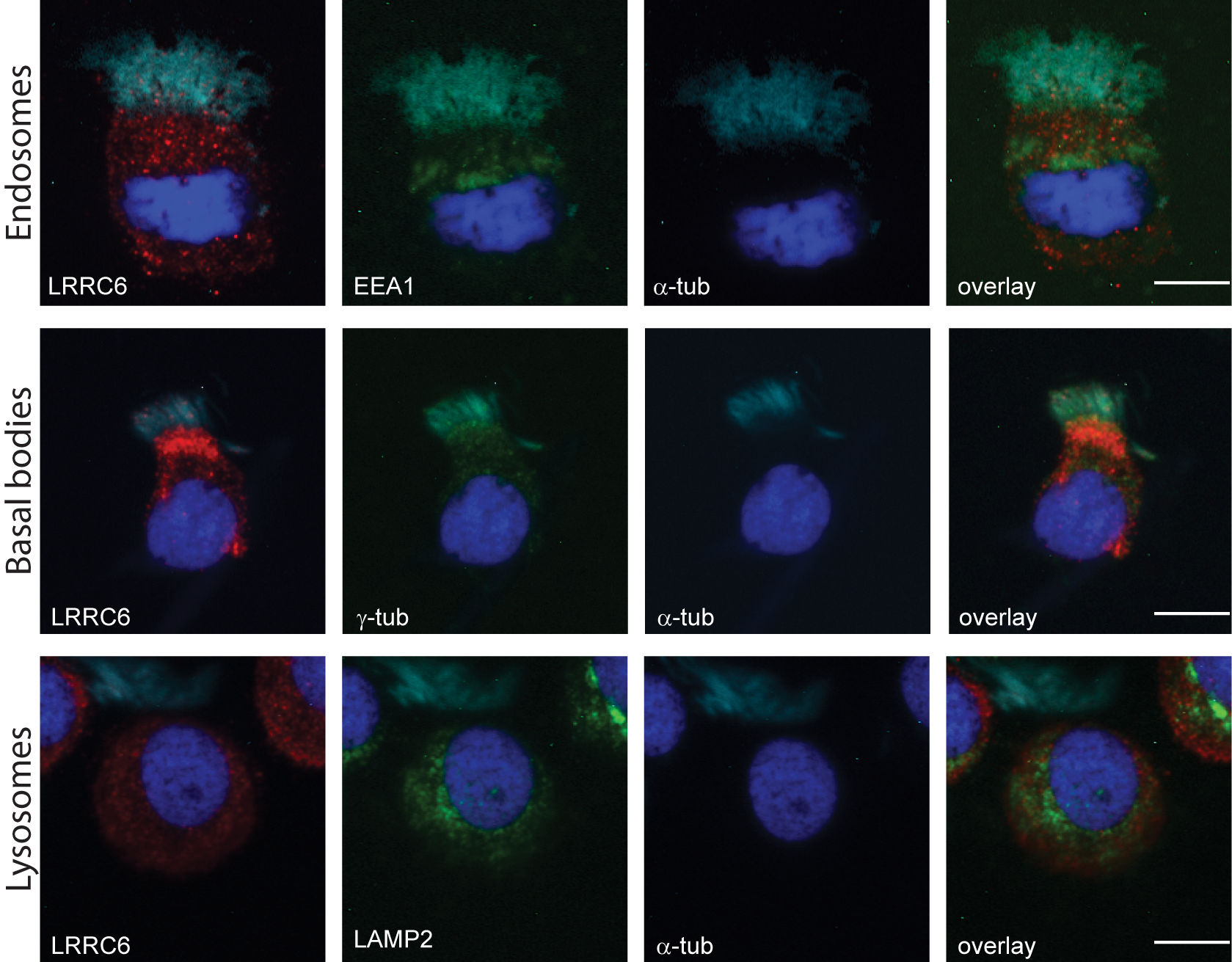

Supplement: Figure S1 — Co-localization of LRRC6 with different organelles. Immunofluorescent staining of tracheobronchial epithelial cells from healthy subject showing no co-localization of LRRC6 (red) with markers of endosomes (green), and lysosomes (green). However, LRRC6 localized with χ-tubulin, a marker for basal bodies (green). Nuclei were stained using DAPI (blue). acetylated α-tubulin, a cilia marker, is shown in turquoise (scale bar = 10 µm). (TIF) [file pone.0059436.s001.tif]

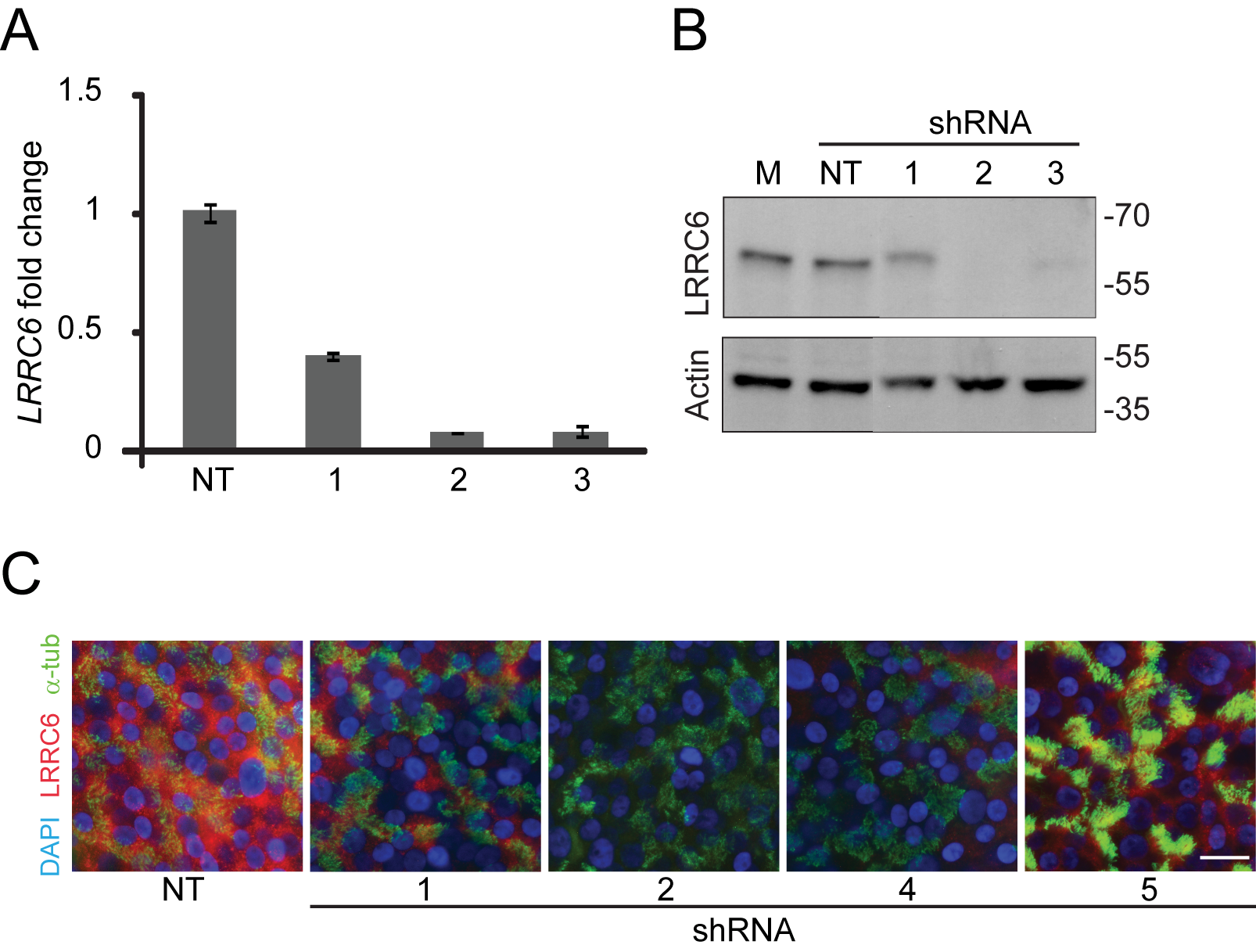

Supplement: Figure S2 — RT PCR analysis of LRRC6 expression in RNAi silenced cells. (A) LRRC6 expression in LRRC6-specific shRNA transfected airway epithelial cells (B) Immunoblot analyses of airway epithelial cells transfected with three different LRRC6-specific shRNA or non-targeted shRNA (NT) sequences and nontransfected control cells (M). (C) En face images of LRRC6 in cultured preparations of ciliated airway epithelial cells from a normal donor, transfected with either non-targeted, control shRNA (NT) or different LRRC6 targeted shRNA sequences. LRRC6 (red), acetylated α-tubulin (green), a ciliated cell marker, and co-stained with DAPI (blue). (scale bar = 20 µm). (TIF) [file pone.0059436.s002.tif]
